# Increased Epicardial Adipose Tissue Is Associated with the Airway Dominant Phenotype of Chronic Obstructive Pulmonary Disease

**DOI:** 10.1371/journal.pone.0148794

**Published:** 2016-02-11

**Authors:** Yuichi Higami, Emiko Ogawa, Yasushi Ryujin, Kenichi Goto, Ruriko Seto, Hiroshi Wada, Nguyen Van Tho, Le Thi Tuyet Lan, Peter D. Paré, Yasutaka Nakano

**Affiliations:** 1 Division of Respiratory Medicine, Department of Internal Medicine, Shiga University of Medical Science, Shiga, Japan; 2 Health Administration Center, Shiga University of Medical Science, Shiga, Japan; 3 Respiratory Care Center, University Medical Center, Ho Chi Minh City, Vietnam; 4 University of British Columbia Center for Heart Lung Innovation, St Paul’s Hospital, Vancouver, BC, Canada; University of Athens, GREECE

## Abstract

**Background:**

Epicardial adipose tissue (EAT) has been shown to be a non-invasive marker that predicts the progression of cardiovascular disease (CVD). It has been reported that the EAT volume is increased in patients with chronic obstructive pulmonary disease (COPD). However, little is known about which phenotypes of COPD are associated with increased EAT.

**Methods:**

One hundred and eighty smokers who were referred to the clinic were consecutively enrolled. A chest CT was used for the quantification of the emphysematous lesions, airway lesions, and EAT. These lesions were assessed as the percentage of low attenuation volume (LAV%), the square root of airway wall area of a hypothetical airway with an internal perimeter of 10 mm (√Aaw at Pi10) and the EAT area, respectively. The same measurements were made on 225 Vietnamese COPD patients to replicate the results.

**Results:**

Twenty-six of the referred patients did not have COPD, while 105 were diagnosed as having COPD based on a FEV_1_/FVC<0.70. The EAT area was significantly associated with age, BMI, FEV_1_ (%predicted), FEV_1_/FVC, self-reported hypertension, self-reported CVD, statin use, LAV%, and √Aaw at Pi10 in COPD patients. The multiple regression analyses showed that only BMI, self-reported CVD and √Aaw at Pi10 were independently associated with the EAT area (R^2^ = 0.51, p<0.0001). These results were replicated in the Vietnamese population.

**Conclusions:**

The EAT area is independently associated with airway wall thickness. Because EAT is also an independent predictor of CVD risk, these data suggest a mechanistic link between the airway predominant form of COPD and CVD.

## Introduction

Cardiovascular disease (CVD) is one of the major causes of death in patients with chronic obstructive pulmonary disease (COPD) [1]. There is considerable evidence that COPD and CVD frequently coexist [2], and each condition affects the prognosis for the other [3]. CVD is the second most common cause of death (after lung cancer) in smokers with mild to moderate airway obstruction [4] and accounts for 27% of the deaths in COPD patients [5]. Therefore, non-invasive markers, which can identify COPD patients at high risk for future CVD, are important.

Epicardial adipose tissue (EAT), which accumulates around the heart between the myocardium and the pericardium, has been shown to be a non-invasive marker for the development of CVD [6]. EAT is considered to play a key role in the pathogenesis of coronary atherosclerosis. It was reported that EAT was associated with CVD risk in the general population [7]. A report from the Multi-Ethnic Study of Atherosclerosis (MESA) indicated that the EAT volume observed by a chest CT could be a predictor of the occurrence of CVD [6]. Further, Zagaceta and colleagues have suggested that the volume of the EAT was higher in COPD patients than in non-COPD patients [8].

We have previously shown that the measurements of airway disease and emphysema assessed by CT are independently associated with aspects of the pathophysiology in COPD [9, 10]. Using these indices, we have also shown that COPD patients can be divided into four phenotypes: an airway-dominant phenotype, an emphysema-dominant phenotype, a mixed phenotype (airway and emphysema), and a normal CT phenotype [11].

However, little is known regarding different risks for CVD among these phenotypes or whether biomarkers of CVD risk, such as the EAT, are more commonly associated with a specific COPD type. The aim of this study was to evaluate the relationship between the CT phenotype and the EAT volume in COPD patients.

## Materials and Methods

### Study Population

**Fig 1:**
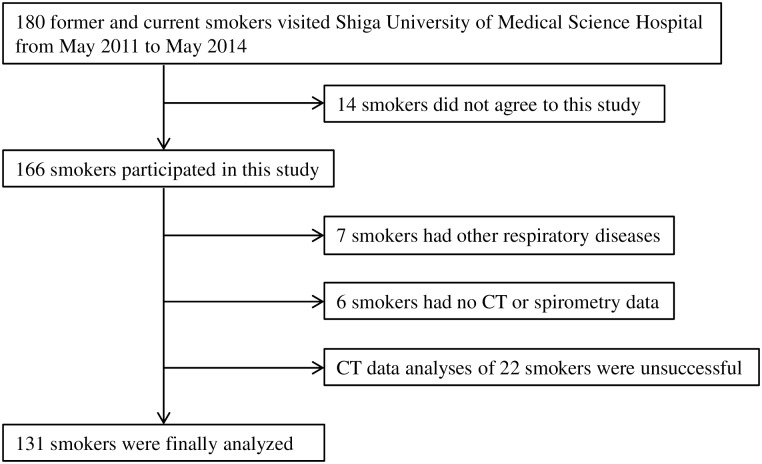
Disposition of Subjects.

One hundred and eighty patients who visited the respiratory medicine outpatient clinic of the Shiga University of Medical Science Hospital from May 2011 to May 2014 were consecutively enrolled in this study. The details of the inclusion and exclusion criteria and the demographic and clinical data for this study are described in S1 Appendix. As shown in Fig 1, 131 subjects from the initial 180 former and current smokers remained in the final analysis. The study was approved by the Ethics Committee of the Shiga University of Medical Science. All participants agreed to participate in the study and provided written informed consents.

### Pulmonary function tests

Spirometry was performed 15 minutes after inhalation of 20 μg of procaterol using a FUDAC-77^®^ spirometer (Fukuda Denshi, Tokyo, Japan) according to the recommendations of the ATS (American Thoracic Society)/ERS (European Respiratory Society) [12]. The predicted values for spirometry were calculated in accordance with the guidelines of the Japanese Respiratory Society [13].

### CT Scans

Chest CT scans were performed using a 320-detector row CT scanner, Aquilion ONE^®^ (Toshiba Medical Systems, Tochigi, Japan). The inspiratory CT images were obtained in the supine position after inhalation of 20 μg of procaterol. The CT images were reconstructed with 1.0 mm slice thickness and 0.5 mm intervals using the FC03 algorithm.

### Quantification of emphysematous and airway lesions

All CT images were analyzed using the Apollo^®^ software (VIDA diagnostics, Coralville, IA, USA) (http://www.vidadiagnostics.com) [14]. Emphysematous lesions were assessed as the percentage of low attenuation volume (LAV%): less than -950 Hounsfield Unit (HU) [14]. The airway wall thickness was assessed by measuring the square root of the airway wall area of a hypothetical airway with an internal perimeter of 10 mm (√Aaw at Pi10) as reported previously [15].

### Quantification of EAT

The EAT area at the origin of the left main coronary artery level, which was strongly associated with total EAT volume [16], was measured by manually tracing the pericardium using ImageJ software (Ver.1.47, National Institutes of Health, Bethesda, Maryland, USA) [17]. An image setting was set as follows: window width -230 to -30 HU and window level -130 HU [16], to identify the pixels corresponding to the adipose tissue. The reproducibility of the intra- and inter-observer measurements of EAT area in randomly selected 100 subjects were fairly well by Bland-Altman analyses (data not shown).

### Measurement of subcutaneous fat area

The subcutaneous fat area (SFA) at the level of the bottom of the right shoulder blade, which was associated with abdominal visceral fat [11], was calculated using ImageJ software (Ver.1.47) [17].

### Coronary artery calcium score

The coronary artery calcium (CAC) scores were evaluated using the Agatston method [18] and are shown in S2 Appendix. The reproducibility of the intra- and inter-observer measurements of CAC scores in randomly selected 20 subjects were very well by Bland-Altman analyses (data not shown).

### Vietnamese COPD patients

Two hundred and twenty-five Vietnamese COPD patients were investigated in this study. The details of the patient characteristics and the methodology for the CT measurements are described in a previous report and are included in S3 Appendix [19]. The protocol for this study was approved by the Biomedical Ethics Committee of University Medical Center in Ho Chi Minh City. Written informed consent was obtained from all Vietnamese subjects.

### Statistical analysis

The statistical analyses were carried out using the JMP software ver. 11 (SAS Institute, Cary, NC, USA). The results for continuous variables are shown as the mean ± standard deviation (SD). The Wilcoxon Rank-Sum test was used to compare groups. Spearman’s rank correlation coefficient was used to evaluate the relationships between the EAT area, SFA and the CT parameters. Bland-Altman analysis was performed to evaluate the reproducibility for the EAT area and CAC scores. A p value less than 0.05 was considered significant.

## Results

### Subject characteristics

Twenty-six non-COPD smokers and 105 COPD patients were enrolled in this study. The characteristics of the subjects are summarized in Table 1. The COPD patients were older, but there was no significant difference in the BMI between the two groups. By definition, the pulmonary function tests in the COPD patients showed substantially obstructed lung function. As expected, the MRC dyspnea scale was significantly higher in COPD patients. The frequency of self-reported CVD was significantly higher in the COPD patients (p = 0.049), but the frequencies of hypertension and diabetes mellitus (DM) were not different between the two groups. There were no significant differences in the oral corticosteroid and the statin use between the two groups.

**Table 1:** Subject characteristics.

	non-COPD (n = 26)	COPD (n = 105)	p value
**Age (years)**	67.0 ± 9.01	73.1 ± 7.51	0.003
**Male (%)**	92.3	92.4	0.990
**BMI (kg/m**^**2**^**)**	23.0 ± 3.25	23.1 ± 2.76	0.738
**Current smoker (%)**	38.5	19.1	0.035
**Pack-Years**	47.5 ± 28.0	61.2 ± 31.4	0.012
MRC dyspnea scale*	1.16 ± 1.25	1.81 ± 1.23	0.022
**FVC % predicted (%)**	100.5 ± 14.7	101.0 ± 18.0	0.716
**FEV**_**1**_**% predicted (%)**	91.6 ± 12.8	66.9 ± 22.3	<0.0001
**FEV**_**1**_**/FVC (%)**	74.3 ± 3.49	52.1 ± 12.8	<0.0001
**DL**_**CO**_ **/ V**_**A**_ (mL/min/mmHg/L)*	3.73 ± 1.02	2.69 ± 0.98	<0.0001
**Self-reported hypertension (%)**	34.6	37.1	0.130
**Self-reported DM (%)**	15.4	20.0	0.118
**Self-reported CVD (%)**	3.85	14.3	0.049
**Oral corticosteroid use (%)**	0.0	6.1	0.196
**Statin use (%)**	19.2	21.9	0.766

The data are presented as the mean ± standard deviation or %.

BMI, body mass index; MRC, Medical Research Council; FVC, forced vital capacity; FEV_1_, forced expiratory volume in 1 s; DL_CO_, carbon monoxide diffusing capacity; DM, diabetes mellitus; CVD, cardiovascular disease.

* COPD (n = 103)

### Quantitative analyses of emphysematous and airway wall lesions on chest CTs

As shown in Table 2, both the LAV% and √Aaw at Pi10 were significantly higher in the COPD patients than in the non-COPD smokers. In the COPD patients, the LAV% was significantly correlated with the carbon monoxide diffusing capacity (DL_CO_/V_A_) (ρ = -0.626, p = <0.0001) and the BMI (ρ = -0.373, p = <0.0001), and the √Aaw at Pi10 was significantly correlated with the BMI (ρ = 0.201, p = 0.039) (Table 3).

**Table 2:** Comparison of the CT-measured parameters between the non-COPD and COPD groups.

	non-COPD (n = 26)	COPD (n = 105)	p value
**LAV% (%)**	3.90 ± 4.48	12.5 ± 11.6	<0.0001
**√Aaw at Pi10 (mm)**	3.70 ± 0.08	3.75 ± 0.08	0.012
**EAT area (cm**^**2**^**)**	10.9 ± 7.69	13.1 ± 6.07	0.038
**EAT area / BMI**	0.45 ± 0.26	0.55 ± 0.22	0.030
**SFA (cm**^**2**^)*	76.0 ± 39.0	79.3 ± 34.8	0.684
CAC score**	1771 ± 3750	1428 ± 2704	0.796
**CAC score >400 (%)** **	46.2	45.6	0.962

The data are presented as the mean ± standard deviation or %.

LAV%, percentage of low attenuation volume; √Aaw at Pi10 (mm), square root of airway wall area of a hypothetical airway with an internal perimeter of 10 mm; EAT, epicardial adipose tissue; SFA, subcutaneous fat area; CAC, coronary artery calcium.

* non-COPD (n = 23) COPD (n = 88)

** COPD (n = 103)

**Table 3:** Spearman’s rank correlation coefficients (ρ/p value) for LAV% and √Aaw at Pi10 in COPD patients.

	LAV%		√Aaw at Pi10	
	ρ	p value	ρ	p value
**Age (years)**	0.077	0.434	0.338	<0.0001
**BMI (kg/m**^**2**^**)**	-0.373	<0.0001	0.201	0.039
**Pack-Years**	0.265	0.006	-0.049	0.618
**MRC dyspnea scale**	0.272	0.006	0.160	0.107
**FVC % predicted (%)**	-0.087	0.378	-0.137	0.163
**FEV**_**1**_** % predicted (%)**	-0.450	<0.0001	-0.149	0.129
**FEV**_**1**_**/FVC (%)**	-0.565	<0.0001	-0.135	0.168
**DL**_**CO**_**/V**_**A**_ **(mL/min/mmHg/L)**	-0.626	<0.0001	-0.049	0.626
**√Aaw at Pi10 (mm)**	-0.072	0.465	-	-

BMI, body mass index; MRC, Medical Research Council; FVC, forced vital capacity; FEV_1_, forced expiratory volume in 1 s; DL_CO_, carbon monoxide diffusing capacity; LAV%, percentage of low attenuation volume; √Aaw at Pi10, square root of airway wall area of a hypothetical airway with an internal perimeter of 10 mm.

### Quantitative analyses of adipose tissue in chest CTs

The EAT area was significantly higher in the COPD group (Table 2). On the other hand, the SFA showed no significant difference between the two groups.

**Fig 2:**
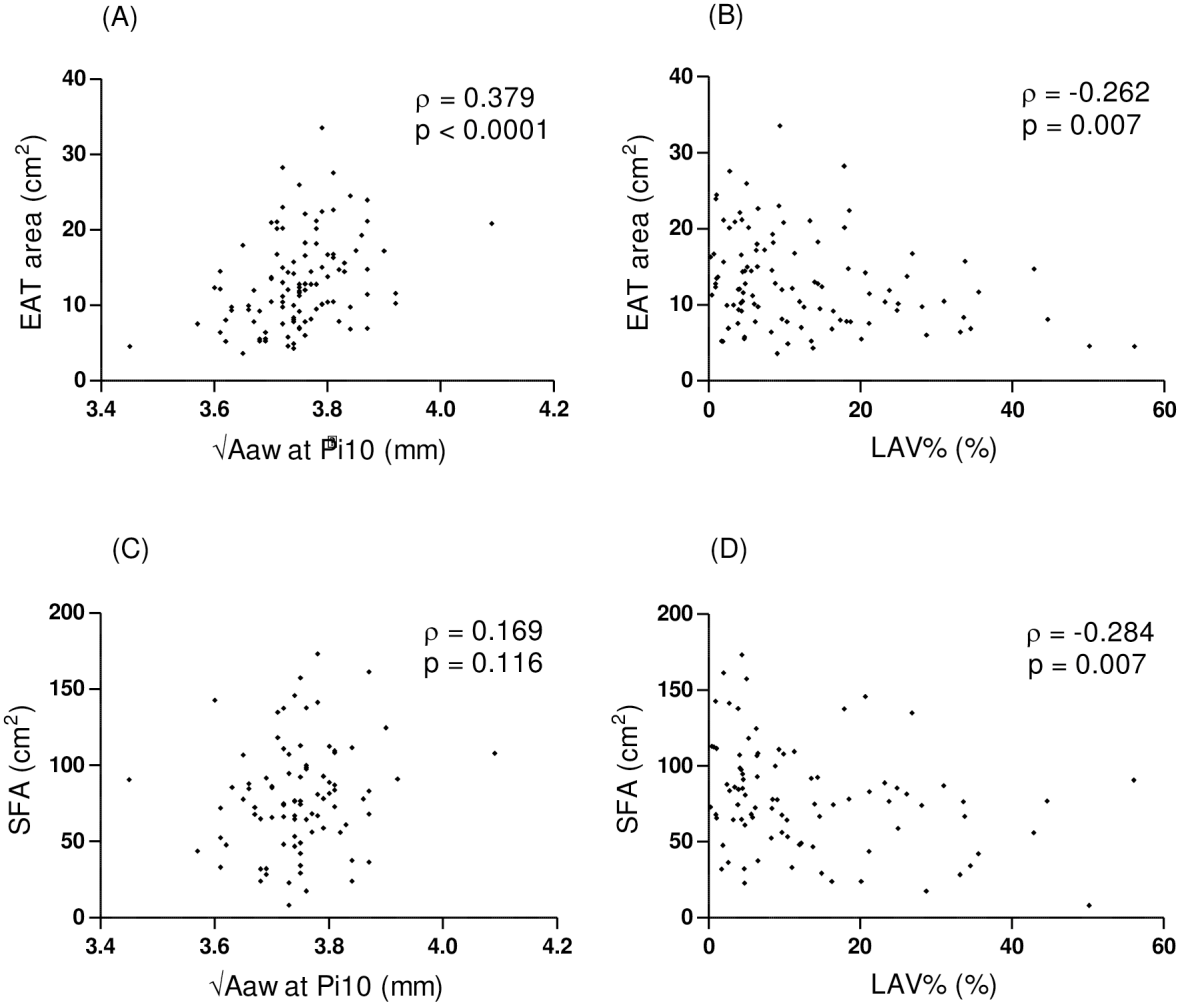
Correlation between adipose tissue and quantitative CT parameters in COPD patients. Spearman’s rank correlation coefficient was used to evaluate these relationships. The EAT area was positively associated with the √Aaw at Pi10 (A) and inversely associated with the LAV% (B). The SFA was inversely associated with the LAV% (D) but not with the √Aaw at Pi10 (C).

In the COPD patients, the EAT area was positively associated with the airway wall thickness, √Aaw at Pi10 (ρ = 0.379, p = <0.0001), and negatively associated with the LAV% (ρ = -0.262, p = 0.007) (Fig 2). The SFA was negatively associated with the LAV% (ρ = -0.284, p = 0.007) but was not related to the √Aaw at Pi10 (ρ = 0.169, p = 0.116) in the COPD patients. Table 4 shows the results of the univariate analyses elucidating the association of the clinical parameters with the EAT area in the COPD patients. The EAT area was significantly correlated with age, BMI, FEV_1_% predicted, and FEV_1_/FVC. An increase in the EAT area was also associated with self-reported hypertension, self-reported CVD and statin use (data not shown). Multiple regression analyses showed that the BMI, self-reported CVD and √Aaw at Pi10 were independent predictors of the EAT area in the COPD patients (R^2^ = 0.51, p < 0.0001) (Table 5).

**Table 4:** Spearman’s rank correlation coefficient (ρ/p value) for EAT area in COPD patients.

	EAT area (cm^2^)	
	ρ	p value
**Age (years)**	0.237	0.015
**BMI (kg/m**^**2**^**)**	0.600	<0.0001
**Pack-Years**	0.071	0.472
**MRC dyspnea scale**	-0.047	0.634
**FVC %predicted (%)**	0.051	0.604
**FEV**_**1**_** %predicted (%)**	0.197	0.044
**FEV**_**1**_**/FVC (%)**	0.234	0.016
**DL**_**CO**_ **/ V**_**A**_ **(mL/min/mmHg/L)**	0.154	0.120

EAT, epicardial adipose tissue; BMI, body mass index; MRC, Medical Research Council; FVC, forced vital capacity; FEV_1_, forced expiratory volume in 1 s; DL_CO_, carbon monoxide diffusing capacity.

**Table 5:** Multiple regression analyses for predictors of EAT area.

	Coefficient	Standard Error	95% Confidence Interval	p value
**BMI (kg/m**^**2**^**)**	1.23	0.15	0.93 to 1.54	<0.0001
**Self-reported CVD (no vs yes)**	-2.64	0.60	-3.82 to -1.45	<0.0001
**√Aaw at Pi10 (mm)**	15.0	5.05	5.03 to 25.1	0.004

EAT, epicardial adipose tissue; BMI, body mass index; CVD, cardiovascular disease; √Aaw at Pi10, square root of airway wall area of the hypothetical airway with an internal perimeter of 10 mm.

### Coronary calcium score and adipose tissue in patients with CVD

**Fig 3:**
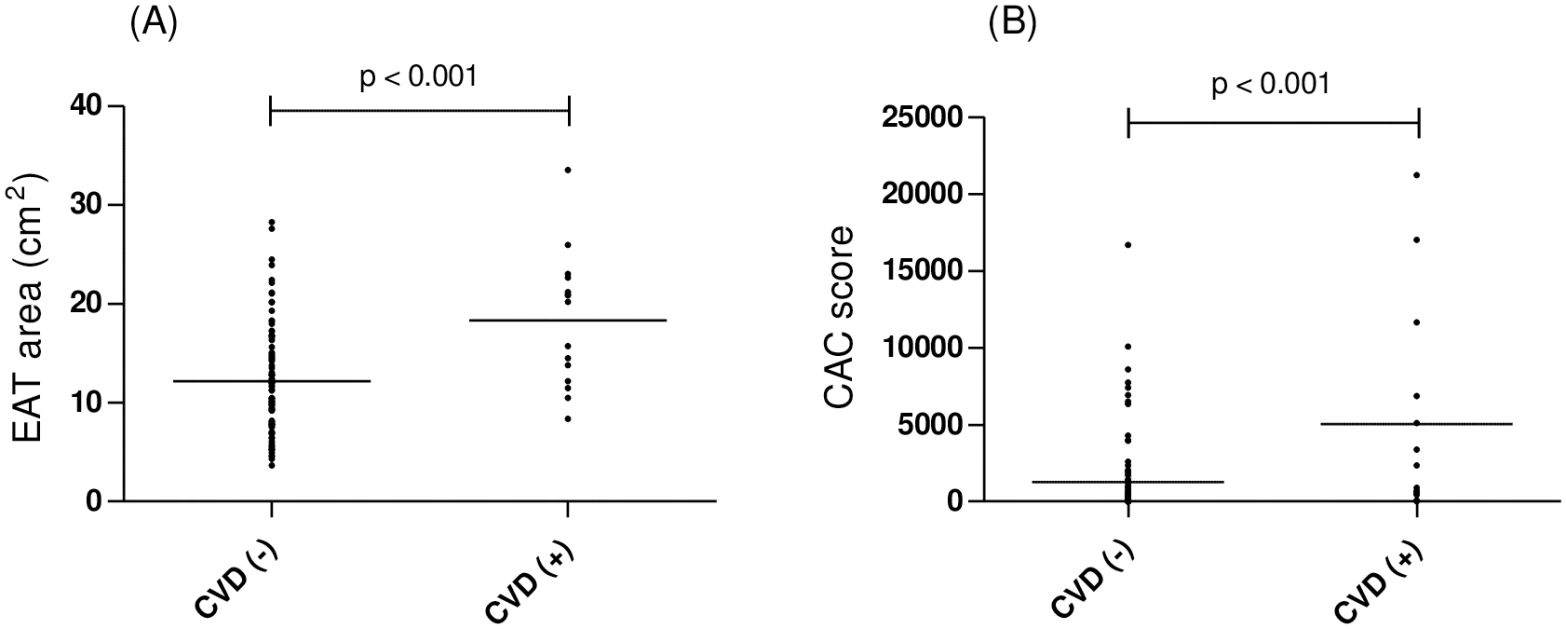
Comparison of EAT area and CAC score between COPD patients with and without CVD. The EAT area (A) was measured using ImageJ software Ver.1.47, and the CAC score (B) was evaluated using the Agatston method. The EAT areas and CAC scores were higher in the COPD patients (p < 0.001).

Unlike the results for the EAT area, there was no significant difference in the CAC scores between the two groups (Table 2). The EAT areas and CAC scores were higher in patients with CVD in the present study (Fig 3), which is consistent with previous studies [6, 20]. Unlike the results for the EAT area, the CAC score was not correlated with the other parameters for the subjects, including age, BMI, pack-years, MRC dyspnea scale, pulmonary function tests, √Aaw at Pi10, or LAV% (data not shown).

### Comparison of data among CT-based phenotypes

**Fig 4:**
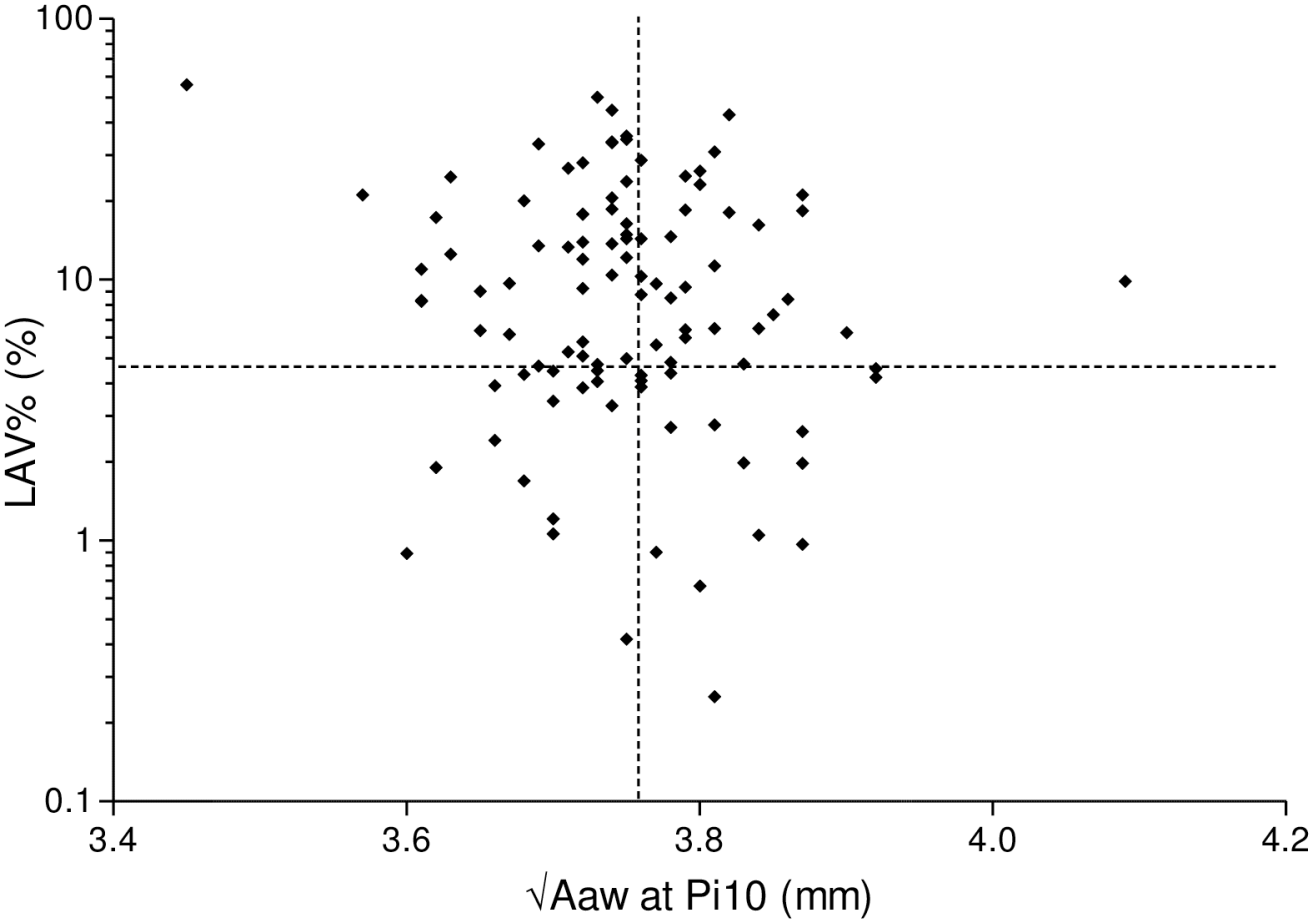
Relationship between √Aaw at Pi10 and the LAV% in 105 COPD patients. The horizontal line shows the third quartile of the LAV% of the non-COPD smokers. The vertical line shows the third quartile of the √Aaw at Pi10 of the non-COPD smokers. Using these cut-off values, the COPD patients can be divided into 4 phenotypes; normal by CT (NCT; low LAV% and low √Aaw at Pi10), airway-dominant (AD; low LAV% and high √Aaw at Pi10), emphysema-dominant (ED; high LAV% and low √Aaw at Pi10) and mixed (Mixed; high LAV% and high √Aaw at Pi10) phenotypes.

We defined upper limits of “normal” for the CT measurements of LAV% and √Aaw at Pi10 as the junction between the 3^rd^ and 4^th^ quartile for these measurements in the non-COPD smokers. Based on these cut-offs, as previously reported [9], the COPD patients were divided into four groups (Fig 4): 1) normal by CT (NCT; low LAV% and low √Aaw at Pi10), 2) airway-dominant (AD; low LAV% and high √Aaw at Pi10), 3) emphysema-dominant (ED; high LAV% and low √Aaw at Pi10) and 4) mixed (Mixed; high LAV% and high √Aaw at Pi10) phenotypes.

The comparisons of the data among the phenotypes are summarized in Table 6. There were no differences in age, pack-years, or MRC dyspnea scale among the four phenotypes. As previously reported, the BMI was significantly lower in the patients with the ED phenotype compared with the AD phenotype [11].

**Table 6:** Classification by CT phenotypes in the COPD patients.

	NCT (n = 16)	AD (n = 16)	ED (n = 43)	Mixed (n = 30)	p value
**Age (years)**	70.1 ± 6.62	74.3 ± 6.87	72.3 ± 8.39	75.4 ± 6.40	0.068
**Male (%)**	87.5	87.5	93.0	96.7	0.595
**BMI (kg/m**^**2**^**)**	23.3 ± 2.34	24.6 ± 2.27†	22.2 ± 2.60	23.4 ± 3.09	0.027
**Pack-Years**	50.3 ± 20.9	52.4 ± 27.7	65.2 ± 29.5	65.8 ± 38.6	0.273
MRC dyspnea scale*	1.50 ± 1.16	2.00 ± 1.26	1.74 ± 1.33	1.93 ± 1.11	0.575
**FVC % predicted (%)**	104.1 ± 13.1	94.0 ± 18.7	102.6 ± 17.3	100.8 ± 20.6	0.360
**FEV**_**1**_** % predicted (%)**	78.3 ± 18.1	69.8 ± 17.4	65.2 ± 22.3	61.6 ± 25.1	0.066
**FEV**_**1**_**/FVC (%)**	59.8 ± 8.54†‡	58.9 ± 8.86†‡	50.0 ± 13.1	47.4 ± 13.3	0.001
**DL**_**CO**_ **/ V**_**A**_ (mL/min/mmHg/L)*	3.19 ± 0.88†‡	3.36 ± 0.81†‡	2.37 ± 0.96	2.56 ± 0.91	0.002

The data are presented as the mean ± standard deviation or %.

BMI, body mass index; MRC, Medical Research Council; FVC, forced vital capacity; FEV_1_, forced expiratory volume in 1 s; DL_CO_, carbon monoxide diffusing capacity.

^†^ p<0.05 compared with the ED phenotype;

^‡^ p<0.05 compared with the Mixed phenotype

* n = 103

**Fig 5:**
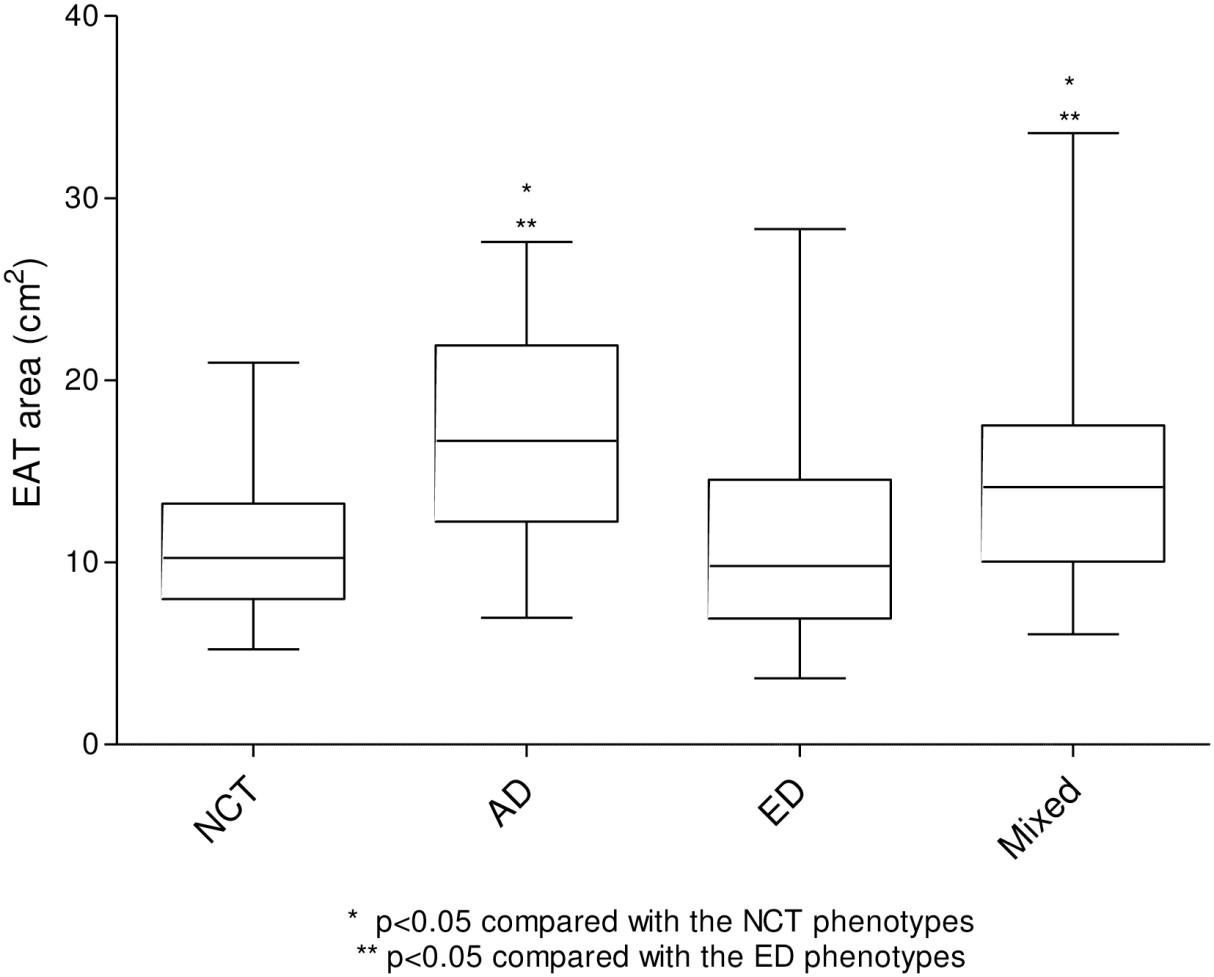
Association of EAT area with the CT phenotypes in COPD patients. The Wilcoxon Rank-Sum test was used to compare groups. The subjects with the AD and Mixed phenotypes had greater EAT areas than those with the NCT and ED phenotypes.

As shown in Fig 5, the subjects with the AD and mixed phenotypes had greater EAT areas than those with the NCT and ED phenotypes. Although there was no significant difference between the patients with the AD and the mixed phenotypes, the EAT area tended to be higher in those with the AD phenotype.

### Vietnamese population

To confirm the above results, the relationships between the EAT area and the CT parameters were investigated in an independent group of 225 Vietnamese COPD patients. The characteristics of these patients are summarized in S1 Table. These subjects were younger and had lower BMIs and smoking histories than the Japanese COPD patients. S2 Table shows the results of the univariate analyses elucidating the associations of the clinical parameters with the EAT areas in the Vietnamese COPD patients. The EAT area was significantly correlated with the BMI, LAV% and √Aaw at Pi10. The multiple regression analyses showed that BMI and √Aaw at Pi10 were independent predictors of the EAT area in the Vietnamese COPD patients (R^2^ = 0.29, p < 0.0001) (S3 Table). Additionally, when subjects were divided into four phenotypes according to previous report [19], the EAT area in those with the AD phenotype was higher than those with the other phenotypes (S4 Table). These results replicate those of the Japanese COPD patients.

## Discussion

In the present study, the subcutaneous fat mass and EAT were separately evaluated using a chest CT, and the relationships of these variables with COPD phenotypes were determined. The epicardial fat accumulation was independently associated with the airway wall thickness in the COPD patients. The EAT area was also associated with the frequency of self-reported CVD. To our knowledge, no previous report has explored the relationship between the epicardial fat area and the COPD phenotype using a chest CT.

EAT, one form of visceral adipose tissue (VAT), produces adipocytokines that play key roles in the progression of atherosclerosis and CVD [21]. Furthermore, EAT may affect the development of CVD more than VAT because it deposits around the coronary arteries. In fact, it has been reported that the CT-measured EAT volume showed a stronger correlation with CVD than abdominal VAT in non-obese patients [22, 23].

Both the EAT and the subcutaneous fat mass were positively correlated with BMI. However, only the EAT was positively correlated with the airway wall thickness. As we have previously reported [11], the BMI was significantly lower in the patients with the ED phenotype. Indeed, in Japanese COPD patients, emphysema is the most common phenotype; the ED and the mixed phenotypes represent the majority of cases [24], Similarly, most of the patients in this study were non-obese (Table 1), but the EAT correlated positively with the √Aaw at Pi10, which suggests epicardial fat accumulation and airway remodeling may share a pathogenetic mechanism. Further evidence that there may be a relationship between airway remodeling and fat metabolism is provided by the fact that subjects with the mixed phenotype, despite having worse lung function than the ED group, did not have a decreased BMI while the emphysema-dominant group did. This result supports our hypothesis that there is a relationship between airway wall thickening and fat accumulation.

Emphysema represents parenchymal destruction, and airway remodeling represents accumulation of extracellular matrix and mesenchymal cell proliferation in the airways, which is observed as an increase in the airway wall thickness as measured by CT [9, 25]. Several reports have found that there is an increased accumulation of fat in chronic inflammatory diseases, including COPD [26–28]. Specifically, it is thought that chronic systemic inflammation contributes to epicardial fat accumulation [8]. Several studies have found associations between increased VAT and chronic inflammatory diseases, including psoriasis [28] and rheumatoid arthritis [29], which suggests specific roles for VAT in inflammation. Two studies have reported that deposition of excessive visceral fat mass was observed by CT in COPD patients [26, 30]. Kishida et al. reported that abdominal visceral fat is more closely associated with the risk factors for CVD than subcutaneous fat [31]. Moreover, increased visceral fat was positively associated with serum IL-6 levels [30]. The reason why EAT is associated with airway wall thickness is unclear. The systemic inflammation is thought to play an important role in the mechanism. However, in this study, we did not measure inflammatory markers. Further studies are needed to clarify the relationship between increased airway wall thickening and epicardial fat accumulation.

In this study, the EAT area was significantly increased in the COPD group (p = 0.038), which is consistent with the previous research [8]. On the other hand, Kaplan and colleagues have recently reported that epicardial fat in COPD patients with right ventricular systolic dysfunction was lower than non-COPD subjects [32]. The reason for this discrepancy could be explained by the difference of the subjects’ background. In this study, the GOLD spirometric classification of the COPD patients was 33, 43 and 29 in GOLD 1, 2 and 3/4, respectively, and epicardial fat accumulation was lower as lung function deteriorated (p = 0.076). However, Kaplan et.al. did not mention spirometric data. Since none of the patient had any physical findings related with acute or chronic heart failure, we did not carry out the echocardiography. We could not discuss whether asymptomatic right ventricular systolic dysfunction was latent or not.

There was no significant difference in the CAC score between the two groups. Previous reports have found a higher CAC score in COPD [33]. The reason for this discrepancy might be that although the non-COPD smokers in this study had normal pulmonary function (FEV_1_/FVC > 70%), they were not “healthy smokers.” Some of these patients had respiratory symptoms, and some were severely obese. Furthermore, there were no significant differences in the prevalence of hypertension and DM between the two groups. Therefore, the CAC score could be influenced more by these comorbidities, whereas the EAT area is related to the pathogenesis of COPD. As previously reported [6, 20], both the EAT areas and the CAC scores were higher in patients with CVD and were independent predictors of CVD in the present study, which suggests that our methods were appropriate for measuring the EAT area and the CAC scores.

Interestingly, airway wall thickness was correlated with the EAT area but not with the CAC score in the COPD patients. As discussed above, epicardial fat may influence chronic inflammation in COPD and contribute to an increased risk for CVD, whereas the CAC may increase the risk for CVD through a different mechanism. The CAC score is specific for the evaluation of the calcification of coronary arteries, an early stage of CVD. These data suggest that COPD and CVD may share the same cause, chronic inflammation, but a direct relationship between the airway wall thickness and coronary calcification may not exist.

We confirmed that the EAT area was related to the airway lesions in the Vietnamese population, although the characteristics of the patients in these two groups were dissimilar. This finding suggests that the results are not specific to the Japanese COPD population but are applicable to other racial groups.

There are some limitations to this study. First, the sample size was relatively small. However, the results from two independent populations were concordant, which increases confidence in the findings. Large longitudinal studies are needed to confirm that the EAT is associated with airway wall thickening in COPD patients. Second, the comorbidities were based on the declarations of the patients and were not independently verified. There was no information about sleep apnea syndrome and the subjects’ physical activity which could influence on fat accumulation. Third, the non-COPD smokers who participated in this study were also from one pulmonary clinic. The advantages were that the approaches and protocols for questionnaires, pulmonary function tests, and CT scanning were standardized. However, as discussed above, our group of non-COPD smokers may not represent so-called healthy smokers. Forth, although the measurement of EAT using CT data without echocardiography has been established by Abbara et al [34], we did not carry out the echocardiography to evaluate EAT in this study.

In conclusion, this is the first study to show that the amount of EAT is associated with airway wall thickening, a specific phenotype of COPD. The risk of CVD may be related to the CT phenotypes in COPD patients.

## Supporting Information

S1 Appendix Study Population. (DOCX)

S2 Appendix Coronary artery calcium scores. (DOCX)

S3 Appendix Vietnamese COPD patients. (DOCX)

S1 Table Subject characteristics of the Vietnamese COPD patients. (DOCX)

S2 Table Spearman’s rank correlation coefficient (ρ/p value) for the EAT area in the Vietnamese COPD patients. (DOCX)

S3 Table Multiple regression analyses for predictors of the EAT area in the Vietnamese COPD patients. (DOCX)

S4 Table Classification by CT phenotypes of the Vietnamese COPD patients. (DOCX)
