# Supplementary material for: Increased Epicardial Adipose Tissue Is Associated with the Airway Dominant Phenotype of Chronic Obstructive Pulmonary Disease
Source: PLoS One. 2016 Feb 11;11(2):e0148794. doi: 10.1371/journal.pone.0148794 (PMC4750940; doi:10.1371/journal.pone.0148794)
Supplement: S1 Appendix — (DOCX) [file pone.0148794.s001.docx]

**S1 Appendix**

**Study Population**

The inclusion and exclusion criteria for this study were as follows. The subjects visited our outpatient clinic either because they had respiratory symptoms, such as cough, sputum, and/or shortness of breath or they were suspected of having COPD by their attending physicians based on their smoking history. Only patients older than 40 years old with a smoking history of at least 10 pack-years were included. The diagnosis of COPD was based on the Global Initiative for Chronic Obstructive Lung Disease (GOLD) [[1](#_ENREF_1)]. The non-COPD group was comprised of current and former smokers without airflow obstruction after the inhalation of a bronchodilator. Patients who had other clinical conditions that could affect spirometry or quantitative parameters, such as asthma, bronchiectasis, interstitial lung disease, acute heart failure, lung cancer or previous lung surgery were excluded. The demographic and clinical data included in this study were age, sex, weight, height, medications, smoking history, and self-reported diabetes mellitus (DM), hypertension, and CVD. The degree of dyspnea was assessed using the Medical Research Council (MRC) dyspnea scale [[2](#_ENREF_2)].

**References**

1. Vestbo J, Hurd SS, Agustí AG, Jones PW, Vogelmeier C, Anzueto A, et al. Global strategy for the diagnosis, management, and prevention of chronic obstructive pulmonary disease: GOLD executive summary. Am J Respir Crit Care Med. 2013;187(4):347-65. doi: 10.1164/rccm.201204-0596PP. PubMed PMID: 22878278.

2. Chronic obstructive pulmonary disease. National clinical guideline on management of chronic obstructive pulmonary disease in adults in primary and secondary care. Thorax. 2004;59 Suppl 1:1-232. Epub 2004/03/26. PubMed PMID: 15041752; PubMed Central PMCID: PMC1766028.
