# Supplementary material for: Increased Epicardial Adipose Tissue Is Associated with the Airway Dominant Phenotype of Chronic Obstructive Pulmonary Disease
Source: PLoS One. 2016 Feb 11;11(2):e0148794. doi: 10.1371/journal.pone.0148794 (PMC4750940; doi:10.1371/journal.pone.0148794)
Supplement: S2 Appendix — (DOCX) [file pone.0148794.s002.docx]

**S2 Appendix**

**Coronary artery calcium score**

The coronary artery calcium (CAC) score was determined using the Agatston method [[1](#_ENREF_1)]. Briefly, coronary calcification was defined as an area more than 1 mm^2^ with a CT density higher than 130 HU. On each axial slice, the area of calcification was calculated and then multiplied by a density score (1, 130-199; 2, 200-299; 3, 300-399, 4, ≥ 400 HU). A total CAC score was determined by adding these numbers. The subjects were divided into two groups based on the total CAC score (≤400 and >400) as described in a previous report [[2](#_ENREF_2)].

**References**

1. Agatston AS, Janowitz WR, Hildner FJ, Zusmer NR, Viamonte M, Detrano R. Quantification of coronary artery calcium using ultrafast computed tomography. J Am Coll Cardiol. 1990;15(4):827-32. PubMed PMID: 2407762.

2. Romme EA, McAllister DA, Murchison JT, Van Beek EJ, Petrides GS, Price CO, et al. Associations between COPD related manifestations: a cross-sectional study. Respir Res. 2013;14:129. Epub 2013/11/21. doi: 10.1186/1465-9921-14-129. PubMed PMID: 24251912; PubMed Central PMCID: PMC3840707.
