# Supplementary material for: Increased Epicardial Adipose Tissue Is Associated with the Airway Dominant Phenotype of Chronic Obstructive Pulmonary Disease
Source: PLoS One. 2016 Feb 11;11(2):e0148794. doi: 10.1371/journal.pone.0148794 (PMC4750940; doi:10.1371/journal.pone.0148794)
Supplement: S3 Appendix — (DOCX) [file pone.0148794.s003.docx]

**S3 Appendix**

**Vietnamese COPD patients**

We included female patients but excluded patients with a history of exposure in biomass smoke to ensure the conditions between the two populations were as similar as possible. Data for comorbidities such as diabetes-mellitus, hypertension and CVD were not available.
