# Supplementary material for: Increased Epicardial Adipose Tissue Is Associated with the Airway Dominant Phenotype of Chronic Obstructive Pulmonary Disease
Source: PLoS One. 2016 Feb 11;11(2):e0148794. doi: 10.1371/journal.pone.0148794 (PMC4750940; doi:10.1371/journal.pone.0148794)
Supplement: S1 Table — (DOCX) [file pone.0148794.s004.docx]

| **S1 Table Subject characteristics of the Vietnamese COPD patients** | |
| --- | --- |
|  | **COPD (n=225)** |
| **Age (years)** | 62.0 ± 9.48 |
| **Male (%)** | 99.1 |
| **BMI (kg/m^2^)** | 21.3 ± 3.34 |
| **Current smoker (%)** | 34.2 |
| **Pack-Years** | 36.7 ± 13.1 |
| **MRC dyspnea scale** | 1.51 ± 1.03 |
| **FVC % predicted (%)** | 77.9 ± 18.1 |
| **FEV_1_ % predicted (%)** | 52.5 ± 19.2 |
| **FEV_1_/FVC (%)** | 49.2 ± 10.8 |
| **LAV% (%)** | 4.36 ± 6.31 |
| **√Aaw at Pi10 (mm)** | 3.78 ± 0.14 |
| **EAT area (cm^2^)** | 9.44 ± 6.21 |
| The data are presented as the mean ± standard deviation or %.  BMI, body mass index; MRC, Medical Research Council; FVC, forced vital capacity; FEV_1_, forced expiratory volume in 1 s; LAV%, percentage of low attenuation volume; √Aaw at Pi10, square root of airway wall area of the hypothetical airway with an internal perimeter of 10 mm; EAT, epicardial adipose tissue. | |
