# Supplementary material for: Increased Epicardial Adipose Tissue Is Associated with the Airway Dominant Phenotype of Chronic Obstructive Pulmonary Disease
Source: PLoS One. 2016 Feb 11;11(2):e0148794. doi: 10.1371/journal.pone.0148794 (PMC4750940; doi:10.1371/journal.pone.0148794)
Supplement: S2 Table — (DOCX) [file pone.0148794.s005.docx]

| **S2 Table Spearman’s rank correlation coefficient (ρ/p value) for the EAT area in the Vietnamese COPD patients** | | |
| --- | --- | --- |
|  | **EAT area (cm^2^)** | |
|  | **ρ** | **p value** |
| **Age (years)** | -0.039 | 0.566 |
| **BMI (kg/m^2^)** | 0.524 | <0.0001 |
| **Pack-Years** | -0.112 | 0.093 |
| **MRC dyspnea scale** | 0.077 | 0.250 |
| **FVC %predicted (%)** | -0.049 | 0.467 |
| **FEV_1_ %predicted (%)** | 0.007 | 0.914 |
| **FEV_1_/FVC (%)** | 0.058 | 0.386 |
| **LAV% (%)** | -0.227 | <0.001 |
| **√Aaw at Pi10 (mm)** | 0.244 | <0.001 |
| EAT, epicardial adipose tissue; BMI, body mass index; MRC, Medical Research Council; FVC, forced vital capacity; FEV_1_, forced expiratory volume in 1 s; LAV%, percentage of low attenuation volume; √Aaw at Pi10, square root of airway wall area of the hypothetical airway with an internal perimeter of 10 mm. | | |
