# Supplementary material for: Increased Epicardial Adipose Tissue Is Associated with the Airway Dominant Phenotype of Chronic Obstructive Pulmonary Disease
Source: PLoS One. 2016 Feb 11;11(2):e0148794. doi: 10.1371/journal.pone.0148794 (PMC4750940; doi:10.1371/journal.pone.0148794)
Supplement: S3 Table — (DOCX) [file pone.0148794.s006.docx]

| **S3 Table Multiple regression analyses for predictors of the EAT area in the Vietnamese COPD patients** | | | | |
| --- | --- | --- | --- | --- |
|  | **Coefficient** | **Standard Error** | **95% Confidence Interval** | **p value** |
| **BMI (kg/m^2^)** | 0.91 | 0.11 | 0.70 to 1.12 | <0.0001 |
| **√Aaw at Pi10 (mm)** | 7.47 | 2.52 | 2.50 to 12.4 | 0.003 |
| EAT, epicardial adipose tissue; BMI, body mass index; √Aaw at Pi10, square root of airway wall area of the hypothetical airway with an internal perimeter of 10 mm. | | | | |
