# Supplementary material for: Increased Epicardial Adipose Tissue Is Associated with the Airway Dominant Phenotype of Chronic Obstructive Pulmonary Disease
Source: PLoS One. 2016 Feb 11;11(2):e0148794. doi: 10.1371/journal.pone.0148794 (PMC4750940; doi:10.1371/journal.pone.0148794)
Supplement: S4 Table — (DOCX) [file pone.0148794.s007.docx]

| **S4 Table Classification by CT phenotypes of the Vietnamese COPD patients** | | | | | |
| --- | --- | --- | --- | --- | --- |
|  | **NCT (n=51)** | **AD (n=38)** | **ED (n=97)** | **Mixed (n=39)** | **p value** |
| **Age (years)** | 62.6 ± 9.31 | 58.7 ± 10.8* | 63.0 ± 9.13† | 61.8 ± 8.77 | 0.044 |
| **Male (%)** | 98.0 | 97.4 | 100 | 100 | 0.363 |
| **BMI (kg/m^2^)** | 21.1 ± 3.08 | 21.6 ± 2.72 | 21.3 ± 3.59 | 21.2 ± 3.67 | 0.861 |
| **Pack-Years** | 37.2 ± 12.9 | 32.9 ± 13.3 | 39.9 ± 12.8† | 31.9 ± 12.2‡ | 0.004 |
| **MRC dyspnea scale** | 1.27 ± 0.92 | 1.42 ± 0.95 | 1.40 ± 1.07 | 2.18 ± 0.91*†‡ | <0.001 |
| **FVC % predicted (%)** | 82.4 ± 18.6 | 74.8 ± 17.7* | 79.0 ± 16.6 | 72.3 ± 20.1*‡ | 0.022 |
| **FEV_1_ % predicted (%)** | 65.8 ± 19.1 | 53.3 ± 17.3* | 50.6 ± 17.5* | 38.8 ± 13.7*†‡ | <0.0001 |
| **FEV_1_/FVC (%)** | 58.5 ± 6.86 | 52.4 ± 7.90* | 46.7 ± 10.4*† | 39.9 ± 7.97*†‡ | <0.0001 |
| **EAT area (cm^2^)** | 8.27 ± 6.20 | 12.7 ± 6.05* | 8.86 ± 5.98† | 9.29 ± 6.15† | 0.002 |
| The data are presented as the mean ± standard deviation or %.  BMI, body mass index; MRC, Medical Research Council; FVC, forced vital capacity; FEV_1_, forced expiratory volume in 1 s; EAT, epicardial adipose tissue.  *p<0.05 versus NCT; †p<0.05 versus AD; ‡p<0.05 versus ED. | | | | | |
